# Minimal change disease and COVID-19 vaccination: Four cases and review of literature 

**DOI:** 10.5414/CNCS110924

**Published:** 2022-07-21

**Authors:** Preeti Chandra, Marisa Roldao, Cinthia Drachenberg, Paulo Santos, Naoki Washida, Alexander Clark, Bipin Bista, Ryunosuke Mitsuna, Angelito Yango

**Affiliations:** 1 Division of Nephrology, University of Maryland, Baltimore, MD, USA,; 2 Department of Nephrology, Centro Hospitalar do Médio Tejo, Torres Novas, Portugal,; 3 Division of Pathology, University of Maryland, Baltimore, MD, USA,; 4 Department of Nephrology, International University of Health and Welfare, Narita Hospital Narita, Japan, and; 5 Baylor Scott and White All Saints Medical Center, Fort Worth, TX, USA; *Contributed equally to the manuscript

**Keywords:** minimal change disease, nephrotic syndrome, graft versus host disease, glomerular disease, COVID-19 vaccine

## Abstract

There have been multiple reports of the development of de novo or relapse of glomerular diseases after SARS-CoV-2 vaccination. While most of them have occurred with the mRNA vaccines (Pfizer/BioNTech and Moderna/NIAID), there also have been reports associated with the vector vaccines (AstraZeneca/ChAdOx1-S) vaccine and the inactivated vaccines. Minimal change disease (MCD) is one of the more common glomerular diseases noted to have been associated with the COVID-19 vaccination. We report here 4 more cases of MCD occurring in association with the COVID-19 vaccine, 3 were de novo cases, and 1 case had a relapse of MCD. We also review all the 41 cases described thus far in the literature and review potential common pathways activated by the vaccination that play a role in the pathogenesis of MCD.

## Introduction 

There have been more than 11 billion doses of the COVID-19 vaccine administered worldwide thus far. These vaccines are primarily the mRNA vaccines, namely Pfizer/BioNTech as well the Moderna/NIAID vaccines, and the vector vaccines; Oxford/AstraZeneca COVID-19 vaccine (ChAdOx1-S (recombinant)) vaccine and Janssen Ad26.COV2.S COVID-19 vaccine. Although the vaccines have been largely well-tolerated and have been life-saving, there have been a small but increasing number of cases of new-onset and relapse of glomerular diseases reported to have occurred in temporal association with the vaccines. In this paper, we report 4 cases of minimal change disease (MCD) that occurred after receiving the COVID-19 vaccine; 3 patients had de novo disease and 1 patient had a relapse of his known MCD which was a manifestation of graft-versus-host disease (GVHD). We also review all the cases of MCD reported in the literature thus far and summarize the findings of those case reports (Table 1). 

## Case reports (Table 2) 

### Patient 1 

A 23-year-old woman with no significant past medical history presented with 5 days of generalized swelling and frothy urine 7 days after receiving her 2^nd^ dose of the Moderna/BioNTech vaccine. She was not taking any prescription or over-the-counter medications and had no history of nonsteroidal anti-inflammatory drug use. She was noted to have 3+ proteinuria on a urine dipstick by primary care and further referred to nephrology. On presentation, she exhibited 3+ bilateral leg edema, mild periorbital edema, an elevated blood pressure of 150/80 mmHg, and a urine dipstick showing 4+ protein. Urine microscopy showed 0 – 2 red cells/HPF with no cellular casts. Initial laboratory testing showed creatinine and albumin levels of 0.6 mg/dL and 2.8 g/dL, respectively, and 24-hour urine protein was 4.1 g. Serological tests for autoimmunity including antinuclear antibodies (ANA), anti-double stranded DNA (ds-DNA), antineutrophil cytoplasmic antibodies (ANCA), and complements (C3, C4) were negative as were test for hepatitis B/C, and HIV. A renal ultrasound showed normal-sized kidneys with no significant abnormalities. A kidney biopsy was performed: contained at least 20 glomeruli with no glomerulosclerosis, necrosis, or crescent formation. Some glomeruli exhibited mild and focal mesangial expansion, but there was no evidence of endocapillary proliferation. Direct immunofluorescence (IF) showed 2+ granular mesangial staining for IgA and C3; rest negative. Electron microscopy (EM) showed patent capillary loops and mild mesangial expansion, but no electron-dense deposits were identified. Podocyte foot processes were diffusely effaced. Based on these histologic findings, the patient was diagnosed with MCD. The patient was started on a 1 mg/kg (70 mg) dose of oral prednisone together with a low dose of furosemide to help control her edema. Complete remission with urine protein-creatinine ratio of < 0.3 g/g and serum creatinine level 0.6 mg/dL was achieved 4 weeks after initiation of the steroid treatment, and tapering of the steroid dosage began at 8 weeks. At the 3-month follow-up, the patient remained in complete remission with creatinine and albumin levels of 0.7 mg/dL and 4.4 g/dL, respectively, and a urine protein-to-creatinine ratio of 0.049 g/g. 

### Patient 2 

A 74-year-old Japanese man, had a medical history that was significant only for hypertension. He was admitted with a 4-week history of edema and a 7-kg increase in body weight. He had received the 2^nd^ injection of the Pfizer/BioNTech vaccine 2 days before the onset of edema. Outpatient medications included amlodipine and irbesartan. Physical findings on admission showed blood pressure, of 116/42 mmHg; the patient had pitting edema of both lower legs. The laboratory findings on admission showed serum albumin, 2.0 g/dL; creatinine, 0.81 mg/dL; estimated glomerular filtration rate, 71.0 mL/min/1.73m^2^ (Modification of Diet in Renal Disease (MDRD) equation). Serological work including tests for ANA, ANCA, complements C3, C4, anti-glomerular basement membrane antibody (anti-GBM), and for hepatitis B, C, and HIV were negative. Urine protein-creatinine ratio was 9.1 g/g, selectivity index value, 0.10. Urine sediment showed no red cells. On the 2^nd^ day of hospitalization, a kidney biopsy was performed. Light microscopy showed no significant glomerular abnormalities, and electron microscopy showed no extensive foot process effacement, deposits, or basement membrane abnormalities. Immunohistochemistry was negative for IgG, IgA, IgM, C1q, C3, C4, κ, λ, or fibrinogen. There were no significant findings on EM. Despite that, it was felt that the patient had MCD since the biopsy was done by the time the patient’s proteinuria was resolving. After admission, the patient’s urine protein-creatinine ratio had decreased to 0.62 g/g, and spontaneous remission from nephrotic syndrome was observed ~ 6 weeks after the onset of symptoms. 

### Patient 3 

A 72-year-old woman with hypertension, obesity, and dyslipidemia who developed foamy urine and peripheral edema 14 days after receiving the 1^st^ dose of the AstraZeneca/ChAdOx1-S vaccine. She had worsening edema, dyspnea, and fatigue over 6 weeks. Baseline renal function was normal (Cr 0.7 mg/dL, normal urinalysis). On admission, her blood pressure was 159/75 mmHg, had diffuse pitting edema, and decreased breath sounds with rales in both lung bases. Laboratory data showed elevated creatinine to 1.8 mg/dL, serum albumin 1.5 g/dL, high cholesterol (326 mg/dL) and triglyceride levels (161 mg/dL). Inflammatory parameters and polymerase chain reaction test for SARS-CoV-2 were negative. Urinalysis showed proteinuria (1,000 mg/dL) with unremarkable urine sediment. 24-hour urinary protein was 5.2 g. A kidney ultrasound suggested normal-sized kidneys with regular shape without hydronephrosis. Chest radiograph demonstrated bilateral pleural infusion. Serologies including ANA, ANCA, anti-GBM, anti-phospholipase A2 receptor antibody, complements C3 and C4, and cryoglobulins were normal. Testing for hepatitis B, C, and HIV was negative. A percutaneous kidney biopsy showed 12 glomeruli: light microscopy was without apparent abnormalities in all glomeruli, interstitium showed small lymphocytic infiltrates, and there was acute tubular injury. IF revealed mild glomerular deposits of IgM (+). EM showed diffuse podocyte foot process effacement. These findings were consistent with MCD and acute tubular injury. Treatment with prednisolone 80 mg daily was initiated. Kidney function remains stable (maximum serum creatinine 2 mg/dL), and peripheral edema gradually improved. The patient was discharged 6 days after initiation of corticosteroid therapy. Two weeks later, serum creatinine was 1.1 mg/dL, serum albumin was 2.5 g/dL, and the urinary protein-creatinine ratio was 0.3 g/g. 

### Patient 4 

A 71-year-old male with prior history of acute myeloid leukemia and subsequent allogeneic hematopoietic stem cell transplantation (HSCT) in 2015, glucocorticoid-induced diabetes who had mild GVHD in the liver following HSCT. He developed nephrotic syndrome in July 2016, renal biopsy was consistent with MCD likely representing GVHD in the kidney, he had complete remission with glucocorticoids and tacrolimus, subsequently was tapered off tacrolimus. He had recurrent nephrotic syndrome in May 2018, repeat renal biopsy had findings similar to the previous biopsy. He required a longer course of tacrolimus and a short course of steroids and achieved remission, he was off tacrolimus in December 2020 and remained on low-dose prednisone 1 mg daily which was stopped in February 2021. His serum creatinine ranged between 1.3 and 1.5 mg/dL, eGFR between 45 and 55 mL/min/1.73m^2^ (MDRD equation). Serum albumin was normal. 

He received the 2^nd^ dose of Moderna/NIAID on April 12, 2021. Within a week of getting the 2^nd^ dose, the patient noted foamy and dark urine, periorbital and leg edema as well as abdominal bloating, he did not seek medical attention then, but labs on April 23, 2021 showed serum creatinine 1.8 mg/dL, serum albumin 2.5 g/dL, and urine protein-to-creatinine ratio was 20 g/g. A subsequent 24-hour urine collection showed protein excretion of 23 g. On physical exam, significant findings included systolic blood pressure in the 150s and generalized pitting edema. A renal biopsy performed in April 2021 showed podocyte, tubular epithelial, and endothelial injury suggestive of GVHD, findings similar to those of the two prior biopsies. Capillary loops were patent without any proliferative, necrotizing, or thrombotic changes. The interstitium showed patchy mononuclear inflammatory infiltrates mainly T cells, rare B cells, there was focal tubular inflammation and degenerative changes in tubules. IF was unremarkable. EM showed extensive effacement of the podocyte foot processes consistent with MCD. There were severe degenerative changes in the endothelium. There were no immune deposits, fibrils, tubuloreticular inclusions, or amyloid. His renal function continued to deteriorate with serum creatinine rising to 3 mg/dL. He received rituximab 1 g infusion on May 6 and May 19, 2021, and a short course of high dose prednisone as well loop diuretics with improvement, and labs ~ 4 weeks later showed serum creatinine to 1 mg/dL, serum albumin 2.3 g/dL, and a urine protein-to-creatinine ratio 2.2 g/g and further decreased to 0.5 g/g as of early December 2021. Peripheral CD19/20 cells remain undetectable as of late December 2021. Given the risk-benefit ratio, especially with the newer COVID-19 variants and the fact that his CD19/20 cells were suppressed, he was advised to take the booster dose. He took the booster on December 12, 2021 and did not notice any change in the color of his urine or foamy urine. Urine protein-creatinine ratio was 0.2 g/g as of late December 2021, serum creatinine in the 1.3 – 1.4 range similar to his baseline before his recent relapse. 

## Discussion and conclusion 

In this report, we describe 4 patients who developed nephrotic syndrome from MCD, occurring 2 – 14 days after the receipt of the COVID-19 vaccine. Three of these patients had de novo disease, 1 patient had a relapse of MCD as a manifestation of renal GVHD. Three of these 4 patients received an mRNA vaccine, and 3 of 4 had symptom onset after receiving the 2^nd^ dose of the vaccine, 3 of 4 had symptom onset within 7 days of receipt of the vaccine. Patient 1 had mild and focal mesangial expansion as well as evidence of IgA deposits on IF. However, there were no electron dense immune deposits on EM which would not be consistent with IgA nephropathy. The IgA deposits on IF likely represent lanthanic deposits in this patient. 

Patients 1 and 3 in our report were treated with high-dose steroids with complete remission (CR) /good response, respectively (patient 3 had CR of proteinuria but creatinine still higher than baseline). Patient 2 attained spontaneous remission without the need for immunosuppression, and there was no podocyte effacement on EM on renal biopsy, likely since the patient’s injury was resolving by the time the patient had the biopsy. Patient 4 had a relapse of MCD likely manifestation of renal GVHD, required rituximab and short-term steroids with significant improvement and eventual CR at 7 months. Patient 4 was also given a booster dose and did not relapse after the booster dose, however, he had persistent suppression of B cells post rituximab at the time of the booster dose which likely lowered his risk for relapse. 

Thus far, there have been 33 case reports documenting 41 patients with MCD occurring after the COVID-19 vaccines (Table 1) [1 – 33]. Reported cases were predominantly in adults, with only 2 cases described in pediatric patients. This difference is likely due to the delayed implementation of mass vaccination in the pediatric population. 

In the 33 case reports comprising 41 patients, 29 (~ 71%) cases occurred after the mRNA vaccine and 9 (~ 22%) cases after vector vaccines. In 7 patients who had symptom onset after the 1^st^ dose, there was also some worsening of proteinuria/recurrent relapse after receiving the 2^nd^ dose. 28 patients (68%) had de novo onset of the disease, and 13 (~ 32%) patients had relapsing MCD. 

Out of the MCD occurring de novo, 24/28 (~ 86%) occurred after the 1^st^ dose of the vaccine, and 4 (~ 14%) occurred after the 2^nd^ dose of the vaccine. Most of the symptoms occurred within a week after the vaccine, though the range varied from 1 to 28 days after the vaccine. In 5 patients in whom the symptoms occurred after the 1^st^ dose, symptoms recurred or worsened when the patient received the 2^nd^ dose, and patient required immunosuppression. 

Out of the 13 reported MCD relapses, 7 (~ 54%) occurred after the 1^st^ dose, and 6 after the 2^nd^ dose. Of the patients with relapses after the 1^st^ dose, 2 of these also had recurrent relapses after the 2^nd^ dose. Of the 13 relapsing cases, 6 were on some form of immunosuppression, mostly low-dose steroids, 5 were not on immunosuppression, and in 2 cases data was not available. Approximately 54% of the relapse occurred from 1 to 8 days after the dose, though the range varied from 1 to 28 days after the dose. 

The diagnosis was made by biopsy in most cases (75%). Immunosuppression was given in almost all cases with a good response. 38/41 (~ 93%) needed immunosuppression, mostly steroids. Three patients required dialysis but were able to come off dialysis with treatment. 

Our case series of 4 patients presents findings similar to the other 41 reports in the literature in that 3 of 4 (75%) occurred were de novo; after an mRNA vaccine; within 7 days of the vaccine dose; and required immunosuppression. Contrary to the literature, most of our patients had symptom onset after the 2^nd^ dose. One patient had spontaneous remission which is uncommon but is described in MCD. Spontaneous improvement was also noted in 2 cases after COVID-19 vaccine after the first dose (Unver et al. [25], Marinaki et al. [28]); however, both times, symptoms relapsed after the 2^nd^ dose, and the patient then required immunosuppression. 

MCD is a rare but known complication that may occur after vaccination, with cases having been reported following administration of tetanus-diphtheria, pneumococcus, influenza, and hepatitis B vaccines [34, 35, 36, 37, 38]. MCD as a manifestation of GVHD is well documented as well [39]. 

As initially proposed by Shalhoub in 1974, T cells are thought to produce a lymphokine that is toxic to the glomerular basement membrane with resultant increased glomerular permeability to albumin. The hypothesis was based on observations including remission of MCD after measles infection (which decreases cell-mediated immunity), the therapeutic benefit of steroids and cyclophosphamide which also decreases cell-mediated immunity, and the occurrence of this disease in patients with Hodgkin’s disease. Subsequent studies have suggested that activation of the T helper 2 pathway in MCD, as well as increased Th17 activity and decreased Treg function in MCD. Additionally proposed mechanisms include activation of co-stimulatory molecules such as CD80 (B7-1) as well as CD40 present on antigen-presenting cells and podocytes, as well as the involvement of B cells and other immunomodulatory molecules [40, 41, 42, 43]. 

In the currently used mRNA vaccines, the mRNA is generated by in vitro transcription process and then encapsulated in a lipid nanoparticle (LNP) for intramuscular delivery. In addition to the desired immune response generated by the viral antigen in the mRNA, there is the potential of stimulating an innate immune response to other non-antigenic components of the vaccine. The various modification and purification processes in mRNA production aim to minimize reactogenicity and optimize immunogenicity [44, 45, 46, 47]. After administration, the mRNA-based vaccine is internalized by antigen-presenting cells, including neutrophils, monocytes, and dendritic cells. The latter two cells are more effective relative to neutrophils in translating mRNA into the antigenic peptide [44, 45]. The mRNA is sensed by toll-like receptors resulting in activation of innate immunity, production of type 1 interferon, maturation of antigen-presenting cells, migration to the vaccine-draining lymph nodes, and activation of adaptive immunity. Excessive immune response and a large amount of type 1 interferon can inhibit the translation of mRNA and reduce the response to the vaccine [44, 45, 46, 47]. The activation of the innate immune response is significantly higher after the 2^nd^ vaccine dose [48]. SARS-CoV-2 mRNA vaccines activate the germinal center-derived B-cell response and production of neutralizing antibodies to the viral spike glycoprotein. The mRNA vaccines are potent inducers of T-cell follicular helper cells. They stimulate a predominantly T helper1 (Th1) response with increased production of Th1 cytokines such as interleukin 2, tumor necrosis factor, and interferon-gamma. The Pfizer vaccine but not the Moderna mRNA vaccine also activated cytotoxic T-cells (CD8) response [44, 45, 46, 47]. 

Similarly, the ChAdOx1 nCoV-19 vaccine has also been demonstrated to elicit primarily a Th1 biased response in addition to neutralizing antibodies [49, 50]. 

The temporal association of relapse of MCD after receipt of the vaccine does not establish causality. However, given the common pathways of innate and adaptive immunity activated by the vaccines and in the pathogenesis of MCD, it is plausible that the vaccine can trigger de novo or relapse of MCD in predisposed individuals. It is interesting to note that in the few cases where MCD occurred after the 1^st^ dose of the vaccine, the symptoms recurred or relapsed after the 2^nd^ dose, further lending support to the association between the vaccine and the MCD relapse. 

Considering billions of vaccine doses have been administered, the number of cases of glomerulonephritis documented thus far is very small, although it is likely that not all cases are being diagnosed or reported. It is likely more reports of vaccine-related glomerular diseases will continue to emerge. It is unclear if the greater number of documented cases with the mRNA vaccines is due to under-reporting of cases associated with the non-mRNA vaccines (viral vector or inactivated vaccines) or reflects a more potent immune-stimulating effect of the mRNA vaccines. 

Given the severe morbidity associated with the COVID-19 viral infection, vaccination is essential, including for all patients with underlying glomerular diseases. As more cases arise, it will be important to create a database of such patients to evaluate the true incidence and to study the predisposing as well as modifiable factors for de novo or relapsing glomerular diseases after the vaccination. This will help guide further management of these patients, for example, if ongoing booster doses are needed. Further research could include a study of biomarkers that can guide the balance between generating an immune response against future viral infection but at the same time avoiding triggering autoimmune illnesses, as well as the possibility of further modification in the mRNA or other vaccines used for these patients with autoimmune diseases. 

## Consent for publication 

Written consent for publication to publish clinical information was obtained from patients and can be made available when requested. 

## Funding 

None of the authors has any source of funding concerning the current manuscript. 

## Conflict of interest 

All the authors declare that they have no competing interests. 

**Table 1.** Published case reports of de novo and relapsing minimal change disease after SARS-CoV-2 vaccines.

Age/ Gen	Vaccine	Dose	Symptoms	MCD	Onset (Days)	Cr (mg/dL)	Alb (g/dL)	Protein (g/day)	UPC (g/g)	Biopsy	Treatment	Outcome	Ref
50/M	Pfizer	1^st^	Edema	D	4	2.3	1.9	6.9	NR	MCD + ATN	CS	CR	Lebedev et al. [1]
34/F	Pfizer	1^st ^ 2^nd^	Proteinuria	R (while on Pred 0.4 mg/kg/d)	10	NR	NR	NR	2.4 after 1^st^ dose. 3 after 2^nd^ dose	NR	CS increased dose	Improvement after 1^st ^ dose. CR after 2^nd^	Kervella et al. [2]
77/M	Pfizer	1^st^	Edema	D	7	2.3	2.5	23.2	NR	MCD + ATN	CS	Persistant renal dysfn, nephrotic proteinuria	D’Agati et al. [3]
65/M	CoronoVac	1^st^	Edema	D	7	1	1.1		11.9	MCD	CS	CR	Dirim et al. [4]
22/M	Pfizer	1^st ^ 2^nd^	Edema Transient proteinuria on IS, no NS	R (while on FK + CS low dose)	2	0.8	2.3	NR	Alb Cr 9.7 g/g	NR	FK + high dose steroids CS+Tac	CR	Schwotzer et al. [5]
61/F	Pfizer	1^st^	Edema	D	1	1.5 Req RRT	2.1	12 (g per L)	NR	MCD	CS	PR	Weijers et al. [6]
33/ F	Inactivated vaccine	2^nd^		R (not on IS)	14	0.6	3	6		–	CS	NR	Ozkan et al. [7]
39/M	Pfizer	1^st^	Edema	R (not on IS)	3	1.8	2.7	8	NR	MCD	CS	CR	Mancianti et al. [8]
80s/M	Pfizer	1^st^	Edema	D	7	1.4	1	15.3	NR	MCD	CS	PR U P/Cr 0.7 g/g	Maas et al. [9]
19/F	ChAdOx1mn0V-19	1^st^	Edema	D	8	1.1	2.1		3.2	MCD Mesangio-proliferative variant	CS	CR	Anupama et al. [10]
60s/M	Pfizer	1^st^	Proteinuria	R (not on IS)	8	0.9	2.8	NR	11.5	NR	CS + CSA	CR	Komaba et al. [11]
63/F	Moderna	1^st^	Edema	D	< 7	1.5	0.7	13.4	NR	MCD + ATN	CS	NR	Holzworth et al. [12]
30/M	Astra Zeneca	1^st^	Proteinuria	R Prior RTX	2	0.9	4.7	NR	1.9	NR	CS	CR	Moorlidge et al. [13]
40F	Astra Zeneca	1^st^	Proteinuria (while on FK + Pred 5 mg qd)	R	1	NR	NR	NR	NR	NR	CS dose increased. Remained on FK	CR	Moorlidge et al. [13]
71/M	Astra Zeneca	1^st^	Anasarca	D	1	10.6 Req RRT	2.8	NR	20	MCD+ ATN	CS	PR	Leclerc et al. [14]
51/M	Janssen	1^st^	Edema	D	7	1.1	1.6	8.6	NR	MCD	CS	Nearing CR	Lim et al. [15]
45/F	Pfizer	1^st^	Edema	D	4	0.8	1.5	8.7	NR	MCD	CS	NR	Abdulgayoom et al. [16]
60/M	Pfizer	1^st^	Edema	D	10	1.3	2.4	NR	5.9 (Alb/Cr)	MCD + ATN	CS	CR	Hanna et al. [17]
43/M	Moderna	1^st^	Edema	D	7	0.9	0.8	15	NR	MCD + IgA	CS	PR	Thappy et al. [18]
15/F	Pfizer	1^st^	Edema	D	4	0.6	1.6	NR	7.7	NA	CS	CR	Nakazawa et al. [19]
83/M	Moderna	2^nd^	AKI	D	28	2.2	2	18	NR	MCD+ ATN	CS	PR	Klomjit et al. [20]
67/F	Moderna	2^nd^	Edema	R (while on Pred 5 mg qd)	21	1.6	2.5	19	NR	MCD	CS + RTX	CR	Klomjit et al. [20]
31/F	Pfizer	2^nd^	Edema	D	21	0.75	0.5	NR	13 g/g	MCD	CS	“good” response	Baskaran et al. [21]
55/M	Astra Zeneca	2^nd^	Edema	D	7	7.2 (peak Cr)	1.8	NR	14.4	MCD+ ATN+ AIN Also on NSAIDS	CS	Improved Cr, proteinuria	Baskaran et al. [21]
75/M	Pfizer	1^st ^ 2^nd^	Edema Edema	D	< 7 7	NR 1.2	NR 1.1	NR 7.7	NR NR	– MCD	NR CS	CR	Kobayashi et al. [22]
33/F	Moderna	2^nd^	Edema	R (IS not stated)	21	NR	2.3	NR	6.4	MCD	NR	NR	Salem et al. [23]
41/F	Pfizer	2^nd^	Edema	D	5	NR	2.6	NR	14.4	MCD	NR	NR	Salem et al. [23]
34/F	Pfizer	2^nd^	Edema	R (IS not stated)	28	NR	2.8	NR	12.9	MCD	NR	NR	Salem et al. [23]
78/M	Pfizer	1^st ^ 2^nd^	Edema Edema	D	4 14	Normal 1.4	NR 1.5	NR 14	NR 12.2	NR MCD	NR CS	NR PR	Dormann et al. [24]
31/F	Janssen	1^st^	Edema	D	1	0.55	1.07	15	15	MCD	CS+RTX	CR	Dormann et al. [24]
67/F	CoronaVac	1^st ^ 2^nd^	Edema Edema	D	7 7	0.6 4.2	2.2 1.5	9 18.6	NR	MCD AIN	Dual RAAS blockade CS + CSA	PR Improved, not PR	Unver et al. [25]
22/M	Pfizer	1^st^	Edema	D	9	NR	1.4	14.4	NR	NR	CS	CR	Nagai et al. [26]
22/M	Astra Zeneca	1^st^	Edema	D	11	0.7	2.3	NR	8.7	MCD	CS	CR	Biradar et al. [27]
55/F	Pfizer	1^st ^ 2^nd^	Edema Edema	D	4 2 days post 2^nd^ dose	0.8 “normal”	2.7 2.2	8.6 12.6		– MCD	Spontaneous remission CS	PR U P/Cr 0.8 at 14 days CR	Marinaki et al. [28]
69/F	Pfizer	1^st ^ 2^nd^	Edema Edema	D	7 < 9 days post 2^nd^ dose	NR 0.65	NR 1.5	NR NR	NR 8	– MCD+focal segmental mesangiolysis	– CS	– CR	Tanaka et al. [29]
42/F	Moderna	2^nd^	Edema	R (not on IS)	11	0.6	4	4.2	3.6	–	CS	CR	Leong et al. [30]
30/M	Pfizer	2^nd^	Edema	R (not on IS)	7	0.7	1.7	0.75	1.3	–	CS	CR	Leong et al. [30]
14/M	Pfizer	1^st^	Edema	D	5	2 Peak Cr 9 Req RRT	2	NR	9	MCD + AIN	CS	PR (Cr normal, P/Cr 0.9)	Jongvilaikasem et al. [31]
51/M	Ad26.COV2	1^st^	Edema	D	7	1.5	1.6	8.6	NR	MCD	CS	CR	Lim et al. [32]
80s F	Pfizer	1^st^	Edema	D	2	3.5	2.2	18.2	Ab/Cr 7.7	MCD + AIN	CS	CR of proteinuria Improved Cr	Hartley et al. [33]
40s/M	Pfizer	1^st^	Edema	R (prior CYC, on Pred 10 qd)	1	1.2	2.6	NR	Alb/Cr 7	–	CS+CSA	CR	Hartley et al. [33]

Cr = creatinine; Alb = albumin; UPCR = urine protein creatinine ratio; F = female; M = male; CR = complete remission; PR = partial remission; D = de novo; R = relapse; NR = non-reported; MCD = minimal change disease; ATN = acute tubular injury; AIN = acute interstitial nephritis; IS = immunosuppression, CS = corticosteroids, FK = tacrolimus, CSA = cyclosporine, RTX = rituximab, CYC = cyclophosphamide. All labs are the values reported during the initial presentation of the patient unless stated otherwise.

**Table 2.** Patients clinical characteristics.

Patient	Age/ Gen	Vaccine	Dose	Symptoms	MCD	Onset (days)	Cr (mg/dL)	Alb (g/dL)	Protein (g/day)	UPC (g/g)	Biopsy	Treatment	Outcome
1	23/F	Moderna	2^nd^	Edema	D	7	0.6	2.8	4.1	NR	MCD IgA mesangial staining but no immune deposits on EM	CS	CR
2	74/M	Pfizer	2^nd^	Edema	D	2	0.8	2	NR	9.1	MCD EM normal but proteinuria already resolving by time of biopsy	Supportive therapy	CR
3	72/F	Astra Zeneca	1^st^	Edema	D	14	1.8	1.5	5.2	NR	MCD+ ATN	CS	CR of proteinuria, serum creatinine and albumin improved
4	71/M	Moderna	2^nd^	Edema	R	7	1.8	2.5	23	20	MCD (GVHD)	CS + RTX	PR at 4 months, CR at 7 months

Cr = creatinine; Alb = albumin; UPC = urine protein creatinine ratio; F = female; M = male; CR = complete remission; PR = partial remission; NR = not reported; D = de novo; R = relapse; MCD = minimal change disease; ATN = acute tubular necrosis; CS = corticosteroids; RTX = rituximab.
